# Studying specificity in protein–glycosaminoglycan recognition with umbrella sampling

**DOI:** 10.3762/bjoc.19.144

**Published:** 2023-12-19

**Authors:** Mateusz Marcisz, Sebastian Anila, Margrethe Gaardløs, Martin Zacharias, Sergey A Samsonov

**Affiliations:** 1 Faculty of Chemistry, University of Gdańsk, Gdańsk, Poland; 2 Intercollegiate Faculty of Biotechnology, University of Gdańsk and Medical University of Gdańsk, Gdańsk, Poland; 3 Physics Department, Technical University of Munich, Garching, Germany

**Keywords:** glycosaminoglycan, molecular docking, protein–glycosaminoglycan interaction specificity, RS-REMD, umbrella sampling

## Abstract

In the past few decades, glycosaminoglycan (GAG) research has been crucial for gaining insights into various physiological, pathological, and therapeutic aspects mediated by the direct interactions between the GAG molecules and diverse proteins. The structural and functional heterogeneities of GAGs as well as their ability to bind specific proteins are determined by the sugar composition of the GAG, the size of the GAG chains, and the degree and pattern of sulfation. A deep understanding of the interactions in protein–GAG complexes is essential to explain their biological functions. In this study, the umbrella sampling (US) approach is used to pull away a GAG ligand from the binding site and then pull it back in. We analyze the binding interactions between GAGs of three types (heparin, desulfated heparan sulfate, and chondroitin sulfate) with three different proteins (basic fibroblast growth factor, acidic fibroblast growth factor, and cathepsin K). The main focus of our study was to evaluate whether the US approach is able to reproduce experimentally obtained structures, and how useful it can be for getting a deeper understanding of GAG properties, especially protein recognition specificity and multipose binding. We found that the binding free energy landscape in the proximity of the GAG native binding pose is complex and implies the co-existence of several binding poses. The sliding of a GAG chain along a protein surface could be a potential mechanism of GAG particular sequence recognition by proteins.

## Introduction

Glycosaminoglycans (GAGs) are long linear periodic anionic polydisperse polysaccharides, with repeating disaccharide units comprised of a hexuronic acid (or galactose in keratan sulfate) and a hexosamine (*N*-acetylglycosamide, GlcNAc or *N*-acetylgalactososamide, GalNAc) throughout a regular alternation of 1→4 and 1→3-glycosidic linkages [1–3]. GAGs are mainly located on the cell surface and in the extracellular matrix [4]. Due to their charged nature, they bind a large amount of water [5]. Although GAGs were previously considered just an inert glue surrounding the cell, GAG research in the past few decades has illustrated the crucial role in cell signaling processes, including regulation of cell growth, proliferation and promotion of cell adhesion, anticoagulation, and wound repair [6–9]. All these processes are mediated through their direct interactions with diverse protein targets such as collagens, chemokines [10–11], and growth factors [12–14], which makes them essential in the cell biology [15–16]. In addition, GAGs also facilitate cell migration, act as shock-absorbers in joints and as a sieve in extracellular matrices and are important in maintaining the compressibility of the cartilage. The participation of GAGs in physiological, pathological, and therapeutic functions results principally from their unique physicochemical and structural features, including high negative charge, high viscosity and lubrication propensities, unbranched polysaccharide structures, low compressibility as well as the ability to attract and imbibe large amounts of water [17].

Unlike proteins or nucleic acids, GAGs are constantly altered by processing enzymes and thus they vary greatly in molecular mass, disaccharide unit composition, and sulfation. Based on their core structure they are categorized into six different classes, viz. heparan sulfate (HS), heparin (HP), hyaluronic acid (HA), chondroitin sulfate (CS), dermatan sulfate (DS), and keratan sulfate (KS). The structural and functional diversities of GAGs are regulated by their sequence, size of the chains, degree of sulfation, and the ability to bind proteins [1,18–21]. This structural diversity of GAGs translates into highly heterogeneous functions and allows them to modulate interactions with various protein molecules in respective biological processes [4]. Most of these interactions are driven by electrostatics and are non-specific in nature, however, some of them are highly specific or selective [22–26].

The structural analysis of GAGs improves the understanding of their biological functions and helps in the development of structure–activity relationships for these important biopolymers [27–28]. Although the composition of the individual saccharide components of GAGs is simple, the structural analysis of GAGs is extremely difficult due to their complex pattern of modification such as epimerization and sulfation [29]. In addition, GAGs’ high flexibility and periodicity render these molecules profoundly challenging to analyze using experimental techniques only [30–31]. Thus, computational approaches could be efficiently used to gain insight into protein–GAG interactions that take place at single-molecule levels [32]. More than a complementary tool, computational approaches provide a better understanding of the role of individual interaction partners (including GAGs, solvent, and ions) by bringing often new and experimentally inaccessible details [33–34]. However, for computational researchers, there are still many challenges to overcome that originate from the physicochemical properties of GAGs, viz. their highly polarized (anionic) nature, their periodicity, and the complexity in decoding their sulfation pattern. Their charged nature necessitates the application of appropriate methods for electrostatics, ions, and solvent, particularly given their abundance in protein–GAG interfaces compared to complexes involving other classes of biomolecules. The periodicity can lead to multipose binding, wherein various configurations of the protein–GAG complex may exhibit similar free binding energies, allowing them to co-exist. Interpreting the “sulfation code”, the amount (net sulfation) and particular positions of the sulfation group (sulfation pattern), could assist in the explanation and prediction of GAG specificity [35]. Computational methodologies like molecular docking and molecular dynamics (MD) have proven to be successful in modelling protein–GAG interactions, particularly examining the fundamental questions related to these interactions such as their specificity, the multipose character of GAG binding and the polarity of the binding poses of these periodic molecules.

In the present work, all-atom MD simulations are conducted to study the dynamics of the protein–GAG complexes, and are complemented by free energy analysis. The free energy analysis of the protein–GAG interactions is important in understanding the nature of the interactions and the stability of the binding pose, including the scenario when several co-existing binding poses are identified. We analyze the binding interactions between the GAGs heparin, heparan sulfate, and chondroitin sulfate, and the proteins basic fibroblast growth factor (PDB ID: 1BFC, https://doi.org/10.2210/pdb1BFC/pdb, [12]), acidic fibroblast growth factor (PDB ID: 2AXM, https://doi.org/10.2210/pdb2AXM/pdb, [13]), and cathepsin K (PDB ID: 3C9E, https://doi.org/10.2210/pdb3C9E/pdb, [36], and PDB ID: 4N8W, https://doi.org/10.2210/pdb4N8W/pdb, [37]). The third complex is known to exist in two different binding poses which are experimentally well established. In this study, the umbrella sampling (US) approach is used to pull away a GAG ligand from the binding site and then pull it back in. The main focus of our study is to evaluate whether the application of the US approach is able to reproduce experimentally obtained structures, and how useful it is for understanding GAG properties as protein recognition specificity and multipose binding. We also check for any trace of transition from the 3C9E to the 4N8W structure by pulling the ligand from its bound position and allowing the ligand to approach the protein from a very distant position to the binding sites.

## Materials and Methods

### Structures and parameters

#### Ligand preparation

GAG structures used in the study consist of two parts: 1. the part from the experimental structure (heparin in the 1BFC [12] and 2AXM [13] complexes and chondroitin sulfate-4 in the case of 3C9E [36]/4N8W [37]), where the length is dp6 (dp stands for degree of polymerization) and 2. an additional part with different degree of sulfation or sulfation pattern (in case of ligands 1 and 2 for 1BFC and 2AXM dp6 desulfated heparan sulfate was added to the reducing end and non-reducing end of the GAG, respectively; in case of ligand 3 for the 1BFC and 2AXM dp6 desulfated heparan sulfate was added both to the reducing and non-reducing end of the GAG; in case of the ligand 4 for the 3C9E/4N8W complex dp6 chondroitin sulfate-6 was added to the reducing end of the GAG. The starting binding mode for the cathepsin K complex with chondroitin sulfate corresponded to the 3CE9 complex. Literature data for the sulfate groups [38] and GLYCAM06 [39] force field parameters were used for GAGs in the subsequent MD simulations. A ^1^C_4_ conformation for the IdoA2S ring was chosen as it was shown to be the essentially dominant conformation in the microsecond scale simulations performed by Sattelle et al. as it is energetically more favorable than the ^2^S_O_ conformation [40].

#### Complex preparation

The obtained ligands were docked using RS-REMD (replica exchange with repulsive scaling), an MD-based docking method [41], to assure proper binding poses of the whole ligand and ring puckering and to be consistent with further simulations. The docked ligands cover the binding site the same way as ligands in the experimental structures. Additionally, since the ligands used in the study are longer, they expand over the binding site and interact with the other parts of the protein as well. Experimental structures cover only a small part of the actual GAG molecule that interacts with the protein (as GAGs are built of tens to thousands of sugar units), therefore using longer ligands does not represent artificial behavior and may provide details of additional naturally occurring interactions. Comparison of the docked poses and PDB structures are presented in Supporting Information File 1, Figure S1.

### MD simulations

All the MD simulations of the complexes obtained by RS-REMD docking were performed in AMBER20 package [42]. A TIP3P truncated octahedron water box with a distance of 20 Å from the solute to the box’s border was used to solvate complexes. Na^+^ counterions were used to neutralize the charge of the system. Energy minimization was performed preceding the production US runs (described in the next paragraph). 500 steepest descent cycles and 10^3^ conjugate gradient cycles with 100 kcal/mol/Å^2^ harmonic force restraint on solute atoms were performed. It was followed by 3 × 10^3^ steepest descent cycles and 3 × 10^3^ conjugate gradient cycles without any restraints and continued with heating up the system to 300 K for 10 ps with harmonic force restraints of 100 kcal/mol/Å^2^ on solute atoms. Then, the system was equilibrated for each window at 300 K and 10^5^ Pa in an isothermal, isobaric ensemble for 100 ps.

US production runs were performed for all of the complexes to pull away ligands from the binding site and then to bring them back to the binding site. US simulations consisted of 40 windows where in each the distance between ligand and the binding site was increased by 1 Å using harmonic restraints with a force constant of 10 kcal/mol/Å^2^. Each window consists of 100 ns of US simulation, therefore each US simulation is 4 μs. Distances between the following atoms were chosen as a reaction coordinate in the corresponding complexes: Cα@Leu225-O5@12IdoA(2S) (the GAG sequence numbering is according to the AMBER order, from reducing to non-reducing end and @ means that a particular atom belongs to a particular residue) for basic FGF-ligand 1; Cα@Leu225-O5@1GlcNS(6S) for basic FGF-ligand 2; Cα@Gly275-O5@6IdoA(2S) for basic FGF-ligand 3; Cα@Gly5-O5@12IdoA(2S) for acidic FGF-ligand 1; Cα@Gly5-O5@1GlcNS(6S) for acidic FGF-ligand 2; Cα@Gly5-O5@4IdoA(2S) for acidic FGF-ligand 3; Cα@Arg296-C3@12GlcA for cathepsin K-ligand 4. The reaction coordinate values increased in each subsequent window, with the starting point for each window taken from the previous one.

The overlap between the probability distributions in adjacent windows was analyzed both using bootstrap error analysis and visually for equilibration and production runs. WHAM (weighted histogram analysis method [43]) was performed using Grossfield’s WHAM program [44] to calculate the potential of mean force (PMF). For bootstrap analysis, 0.001 iteration tolerance, 300 K as temperature, and 1000 as number of Monte Carlo trials were used.

After completing the last window of US simulation, 500 ns unrestrained MD runs were carried out in the same isothermal isobaric ensemble to relax the system. A time step of 2 fs and a cut-off of 8 Å for electrostatics were used. The particle mesh Ewald method for treating electrostatics [45] and SHAKE algorithm for all the covalent bonds containing hydrogen atoms [46] were implemented in the MD simulations. The cpptraj program of AMBER was used for the analysis of the trajectories [47]. In particular, native contacts command with default parameters was used for the analysis of the contacts between protein and GAG molecules established in the course of the simulation.

### Binding free energy calculations

MM/GBSA (molecular mechanics generalized born surface area) model igb = 2 [48] from AMBER20 was used for free energy calculations on the trajectories obtained from RS-REMD simulations.

#### GAG binding pose accuracy evaluation

For the evaluation of the binding pose accuracy RMSD and RMSatd values were used. RMSD stands for root mean square deviation and it is defined as the average distance between the atoms of superimposed molecules. RMSatd (root mean square atom-type distance) is very similar to the widely used RMSD but instead of using specific atoms it compares atom types (e.g., any carbon atom to any carbon atom instead of specifically numbered carbon atom to the carbon atom with the same number). RMSatd is more appropriate when used for long and periodic molecules (such as GAGs), when a shift by one periodic unit yields the same pose but would result in high RMSD. The similar issue happens when GAG is rotated by 180°: although it occupies the same binding site and the pose is similar, the RMSD value would be expressed in tens of angstroms, while the RMSatd value would be significantly smaller.

Data analysis and its graphical representation were done with the R-package [49] and VMD [50].

## Results

**Figure 1:**
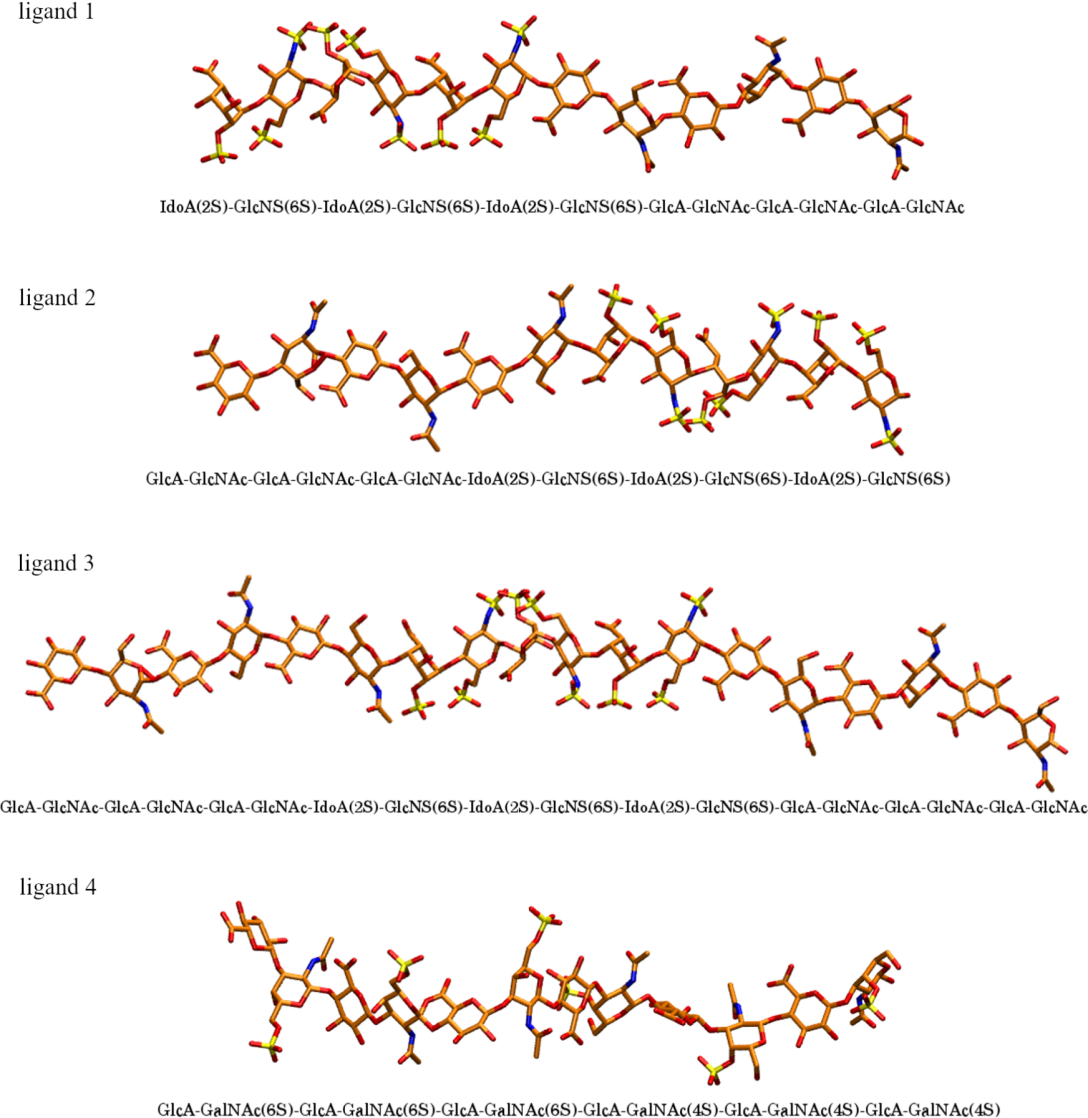
Graphical representation of ligands (in licorice, hydrogen atoms not shown) used in this study. Ligands 1, 2, and 3 were used in complexes with basic fibroblast factor (as modifications of the 1BFC PDB structure) and acidic fibroblast factor (as modifications of the 2AXM PDB structure) while ligand 4 was used with cathepsin K.

In total, 14 US simulations were performed to investigate the specificity of GAG–protein interactions, capabilities of US simulations to dissociate and reassociate protein–GAG complexes in these systems, and potential use of the US simulations in docking of GAG molecules to proteins. In order to do so, six different heparin systems (3 for basic FGF and 3 for acidic FGF) and one chondroitin sulfate system (with cathepsin K) were prepared. For each of the systems 2 US simulations were set up. First, hybrid GAGs (Figure 1) were prepared and docked using RS-REMD to find the pose in the binding site with the lowest interaction energy. Then, the GAG was pulled away from the binding site until it was shifted 40 Å from the starting position. Afterwards, the GAG was pulled in towards the binding site to observe if it reproduces a pose similar to the starting pose. To describe these unbinding and rebinding processes, analyses of RMSD, binding energy, contacts, and hydrogen bonds were performed. Additionally, after the final pulling step, a short MD run of 500 ns was performed to relax the system and to check if the final pose was energetically stable or if it changed during the relaxation step. The data depicted in the graphs result from the analysis of merged US trajectories. While this representation is not entirely physically sound, as the outcomes for each US window reflect the system's state under particular conditions with explicitly defined reaction coordinate values, the visualization of these continuous data potentially offers a more comprehensive insight into the complexity of the system related to its dynamic behavior within each window.

### Basic FGF

**Figure 2:**
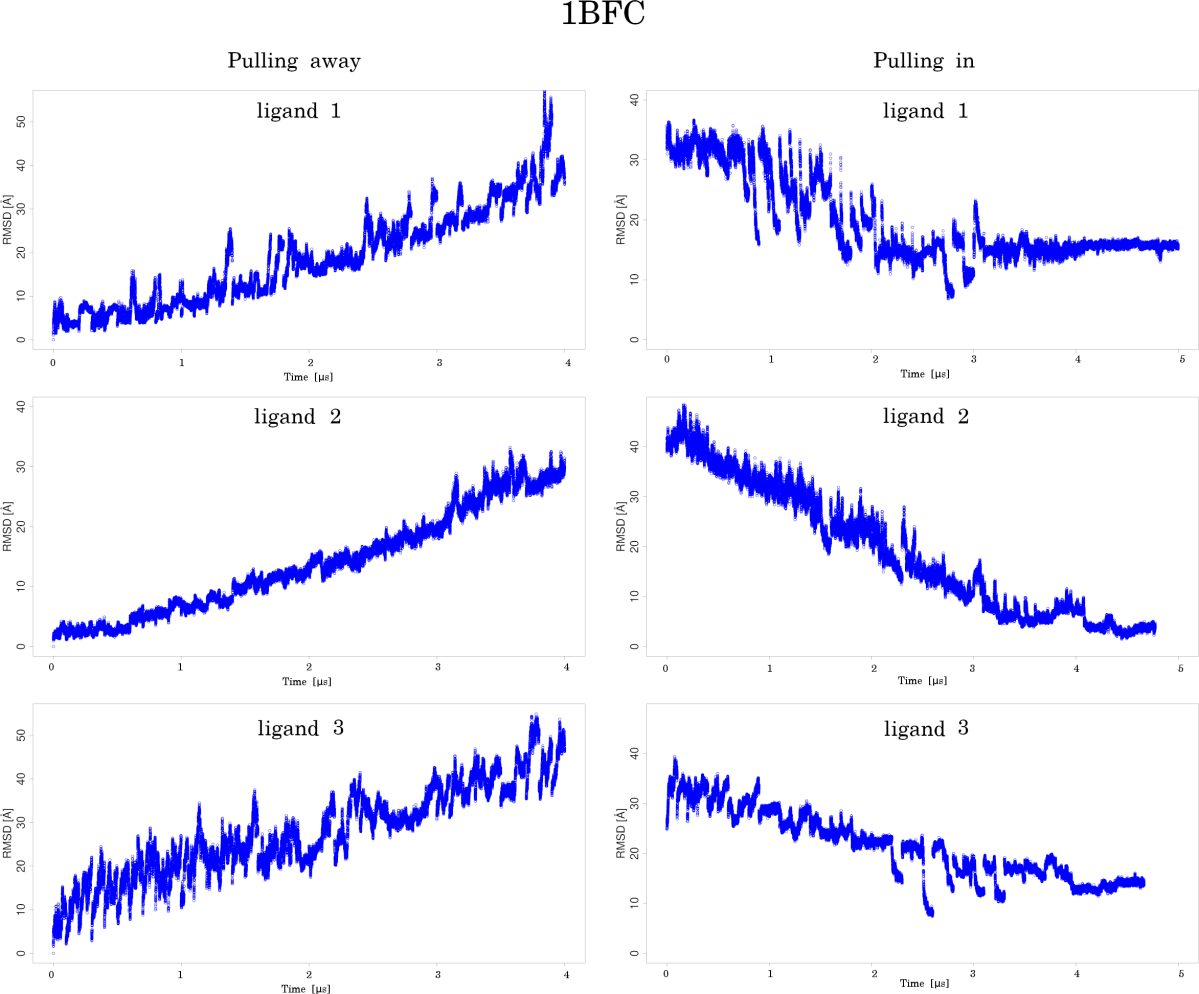
RMSD values obtained using the cpptraj script from AMBER suite for 1BFC (basic fibroblast factor) complexes with 3 different ligands: the ligand is pulled away from the binding site (left panel) and is pulled in towards the binding site (right panel).

**Figure 3:**
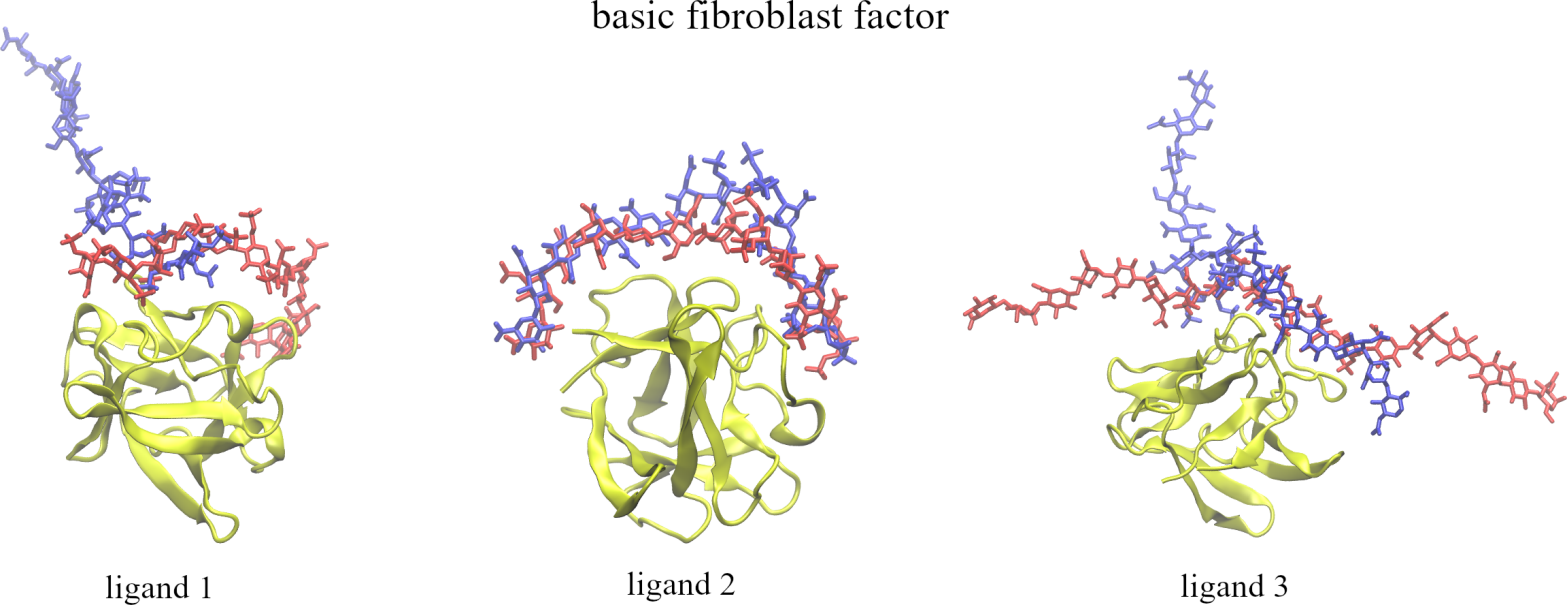
Graphical representation of the ligands’ starting (in red, licorice) and final (in blue, licorice) positions in regard of the binding site of basic fibroblast factor (in yellow, new cartoon).

**Ligand 1.** The RMSD increased gradually up to values of around 40 Å during the unbinding process, and then decreased slowly when it was pulled in. After about the 20th window RMSD stabilized between 15 and 20 Å, suggesting that the GAG did not find the initial pose and was trapped in a different minimum (Figure 2). The same scenario was observed in terms of the binding energy (Supporting Information File 1, Figure S2). When the ligand was pulled away the energy increased and when it was pulled in the energy slowly decreased and converged after about 20 windows. The number of native contacts when the ligand was pulled away rapidly dropped from 1500 to 0 and remained 0 for the rest of the US run (Supporting Information File 1, Figure S3). When pulled in, between 20 and 30% native contacts are restored after the 25th window but not to the original level. Even the additional relaxation MD run did not restore any native contacts. This suggests that the GAG gets close to the binding site but does not return to a similar conformation as the initial (experimental) pose. A similar trend is observed with hydrogen bonds where the number of H-bonds drops when the ligand is pulled away but never gets fully restored after being pulled in to the initial pose. Visual analysis supports the observation that only a small part of the GAG chain from the final pose overlaps with its starting position. The final pose is perpendicular to the initial one (Figure 3).

**Ligand 2.** RMSD slowly increased when pulled away and then when pulled in it gradually decreased to between 6 and 8 Å. During the additional relaxation step, RMSD was further reduced to 3 to 4 Å suggesting that the GAG finds a pose similar to the starting one (Figure 2). The binding energy gradually increased when the ligand was pulled away (from around −150 kcal/mol to around −30 kcal/mol) (Supporting Information File 1, Figure S1). Then, when pulled in, the energy almost did not decrease at the beginning. Only after the 21st window the energy started to decrease more rapidly but it did not go back to the values of −150 kcal/mol corresponding to the initial pose and oscillated around −120 to −100 kcal/mol. During additional relaxation the energies decreased to the range from −130 to −100 kcal/mol. This shows that after an additional MD run, the binding pose did not only become closer to the original structure but also was stabilized energetically in comparison to the pre-relaxation step. The number of native contacts significantly dropped after the first part of US (from ≈2000 contacts to below ≈500) and then stabilized at around 200 to 300 contacts in the last windows (Supporting Information File 1, Figure S3). When the ligand was pulled back to the binding site, only some native contacts were restored (≈500), but during the subsequent relaxation the number of restored native contacts increased to more than 1000. In case of H-bonds, at the end of the US 70 to 90% of them were restored. Visually, both the final and the initial poses look very similar (Figure 3), and this is also reflected in very low RMSD values (3 to 4 Å for such long and flexible molecules is considered to reflect high structural similarity).

**Ligand 3.** Similar to the other ligands, when pulled away from the binding site the RMSD of ligand 3 gradually increased and during pulling in it slowly decreased but did not return to the initial pose which is represented in the RMSD value of 12 Å at the end of the US simulation. Additional relaxation MD also did not result in any significant decrease of RMSD (Figure 2). The initial binding energy of −160 kcal/mol increased very fast at the start of pulling away and finished below −30 kcal/mol at the end (Supporting Information File 1, Figure S2). During the pulling in of the GAG, the energy decreased slowly and reached −70 to −50 kcal/mol at the end of US. However, after the 37th window the binding energy drops below −120 kcal/mol suggesting a more favorable novel ligand conformation. During relaxation, MD energies only improved slightly which is in agreement with the high RMSD that suggests that GAG did not return to the initial binding pose. The number of native contacts decreased drastically during the first windows of US from 1800 to 0 in the 13th window (Supporting Information File 1, Figure S2). During pulling in ligand towards the binding site only a small percentage of the native contacts were restored (50 to 150 native contacts in the last windows). After the relaxation MD the number of contacts went up to 200 to 250, but it never reached levels close to the initial ones which also suggests that the GAG did not get close to the binding site. The number of H-bonds at the end of pulling in was similar to the start of pulling away. However, none of the H-bonds at the end of the US simulation were established between the same atoms as at the start. Visually, only a part of the GAG’s final pose overlaps with the initial one. The final pose adapts a perpendicular conformation to the starting GAG chain orientation (Figure 3).

### Acidic FGF

**Figure 4:**
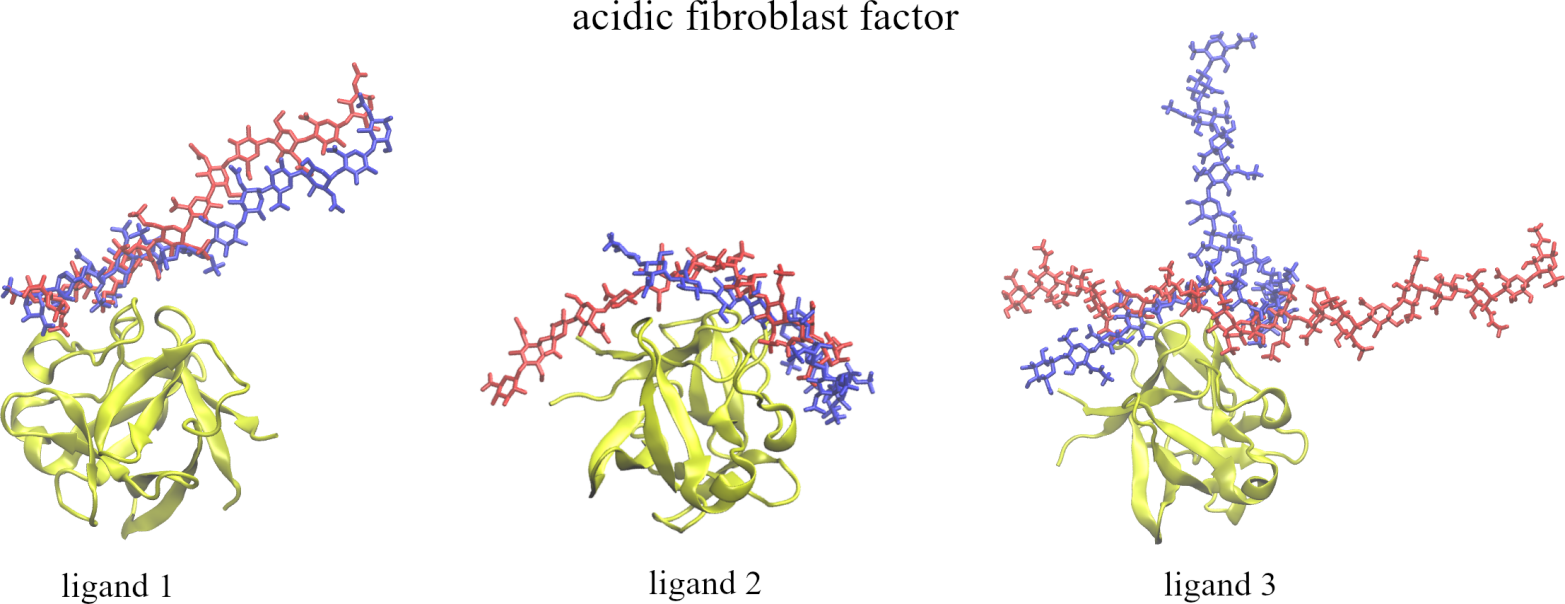
Graphical representation of the ligand’s starting (in red, licorice) and final (in blue, licorice) poses in regard of the binding site of acidic fibroblast factor (in yellow, new cartoon).

**Ligand 1.** RMSD slowly increased during the first phase (windows from 1 to 8) of pulling away and afterwards with the pace similar to the other systems analyzed in this work. On the way back, RMSD of the ligand steadily decreased reaching values 4 to 5 Å at the end of the pulling in (Supporting Information File 1, Figure S4). During the relaxation step, RMSD remained around the same level and did not decrease further. Binding energy started at −140 kcal/mol and increased fast during the first 30 windows (Supporting Information File 1, Figure S5). Afterwards, it oscillated between −20 and 0 kcal/mol. During pulling in of the ligand the energy did not change before window 25 when it started to decrease reaching −90 kcal/mol at the last window. During the relaxation, the energy remained at a similar level. Interestingly, despite the low RMSD at the end, the final energy is less favorable (−90 kcal/mol) than the one observed at the beginning of the US (−140 kcal/mol). The number of native contacts dropped to zero around the 15th window and remained 0 for the rest of the pulling away (Supporting Information File 1, Figure S6). During pulling in, no restoration of native contacts was observed. Also the number of H-bonds when the ligand was pulled all the way in was slightly lower (70 to 80%) than before it was pulled away. The number of H-bonds and native contacts suggest an overall smaller amount of interactions between the ligand and the receptor, but also the establishment of non-native contacts. Visually, the poses from the start and at the end of the US simulations look very similar (Figure 4), with major differences observed around the part of the GAG that is not bound to the protein.

**Ligand 2.** RMSD increased slowly until the 7th window where it started to increase more rapidly. During pulling in, RMSD did not decrease significantly (although visually the ligand is getting close to the initial binding pose) suggesting a drastically different pose of the ligand (Supporting Information File 1, Figure S4). Additional relaxation also did not improve RMSD significantly. In terms of energy of the system it started around −140 kcal/mol and it dropped to the level between −20 and 0 kcal/mol after the 27th window (Supporting Information File 1, Figure S5). During pulling in the energy did not improve significantly. The number of native contacts dropped from ≈1000 to 0 after the 15th window of pulling away (Supporting Information File 1, Figure S6). Only very few native contacts were restored during pulling in at maximum showing 200 of them. The number of H-bonds during pulling in was slightly lower than during pulling away suggesting less interactions between the ligand and the receptor on the way back than at the start of the US simulation. Visually, the major part of the GAG at the end of the US simulation overlapped with its starting pose. However, the final structure is more bent and shifted by about 3 rings relative to the initial one (Figure 4). This is also confirmed by relatively high RMSD values that did not improve much during the course of the pulling in US.

**Ligand 3.** RMSD increased slowly during the first few windows but unlike the other ligands in this particular case the scenario for RMSD did not change significantly afterwards. During pulling in the ligand back to the binding site only a low RMSD decrease was observed (Supporting Information File 1, Figure S4). During the relaxation, again, only a minor decrease in RMSD was observed suggesting that a slightly more favorable pose was achieved. In terms of the energy evolution during pulling away, it started around −140 kcal/mol and then it increased up to the 30th window where it stabilized below 0 kcal/mol (Supporting Information File 1, Figure S5). On the way back, we observe only a partial improvement of the binding energy as it reached values from −80 to −70 kcal/mol at the end of pulling in. However, during relaxation the energy lowered to values from −130 to −120 kcal/mol suggesting binding almost as strong as at the start of the US. The relatively high RMSD and low energy can be justified by the fact that the obtained pose of the ligand was very different from the initial one but there is a small overlapping part that interacts with the ligand around the binding site which can serve as basis for this strong binding. The number of native contacts at the beginning was 1300 and decreased slowly (Supporting Information File 1, Figure S6). In the second part of pulling ligand away changes in number of native contacts were sudden and drastic but they never went completely to 0. The number of contacts oscillated between 50 and 500. On the way back of the ligand, changes are much more subtle and the number of contacts remained between 200 and 400. During the relaxation no significant changes in the number of native contacts was observed. More H-bonds were present at the beginning of pulling the ligand away than at the end of the pulling in suggesting more interactions between the ligand and the protein at the start than at the end of the US. Visually, the final pose of the GAG is much different than the initial one. It is significantly bent and adapts a perpendicular conformation with regard to the starting pose (Figure 4). However, the sulfated part of the GAG overlaps with its initial position.

Additionally, the correlation between the ligand’s RMSD and MM/GBSA per frame was analyzed (Table 1). In all cases positive correlations between analyzed values was observed. However, in some cases this correlation was below 0.5. This is in agreement with the data described above, which showed that despite a significantly different binding pose, sometimes the GAG maintained a relatively strong binding to the protein. This is particularly true for ligand 3 of acidic fibroblast growth factor, which when pulled back into the binding site led to low binding energies but a drastically different pose (partially perpendicular) of the ligand.

**Table 1:** Pearson correlation coefficients between energies obtained from MM/GBSA analysis and RMSD values of the ligand for all frames of the merged MD trajectories.

basic fibroblast growth factor (1BFC)					

ligand 1 (away)	ligand 1 (in)	ligand 2 (away)	ligand 2 (in)	ligand 3 (away)	ligand 3 (in)
0.77	0.60	0.84	0.90	0.80	0.58

acidic fibroblast growth factor (2AXM)					

ligand 1 (away)	ligand 1 (in)	ligand 2 (away)	ligand 2 (in)	ligand 3 (away)	ligand 3 (in)
0.81	0.78	0.63	0.35	0.50	0.25

Energy contributions of sulfated and unsulfated parts of the GAG were investigated from per residue decomposition of MM/GBSA analysis (Table 2). In every case, sulfated parts were always contributing more to the receptor binding than unsulfated ones. Usually, the sulfated part contributed 3–5 times stronger than the unsulfated part. However, during pulling in of ligand 3 for basic fibroblast growth factor the unsuflated part contributed significantly (−7.6 kcal/mol for the unsulfated part in comparison to −10 kcal/mol for the sulfated part, respectively). More interestingly in this case during the pulling in process the contribution of the sulfated part decreased while the one of the unsulfated part increased. This could be interpreted as that the binding of the unsulfated residues can partially compensate the energy loss due to unbinding of the sulfated residues, suggesting rather non-specific interactions between the protein and the ligand.

**Table 2:** Energy contributions in kcal/mol of the sulfated and unsulfated parts of GAGs obtained from MM/GBSA per residue decomposition.

basic fibroblast growth factor (1BFC)											

ligand 1 (away)		ligand 1 (in)		ligand 2 (away)		ligand 2 (in)		ligand 3 (away)		ligand 3 (in)	
sulfated	not sulfated	sulfated	not sulfated	sulfated	not sulfated	sulfated	not sulfated	sulfated	not sulfated	sulfated	not sulfated
−13.1	−4.7	−12.8	−4.6	−16.1	−8.3	−11.9	−6.1	−12.1	−4.7	−10.0	−7.6

acidic fibroblast growth factor (2AXM)											

ligand 1 (away)		ligand 1 (in)		ligand 2 (away)		ligand 2 (in)		ligand 3 (away)		ligand 3 (in)	
sulfated	not sulfated	sulfated	not sulfated	sulfated	not sulfated	sulfated	not sulfated	sulfated	not sulfated	sulfated	not sulfated
−11.4	−1.6	−4.4	−0.7	−11.1	−2.5	−14.0	−6.8	−14.8	−3.6	−7.8	−0.8

### Cathepsin K

**Figure 5:**
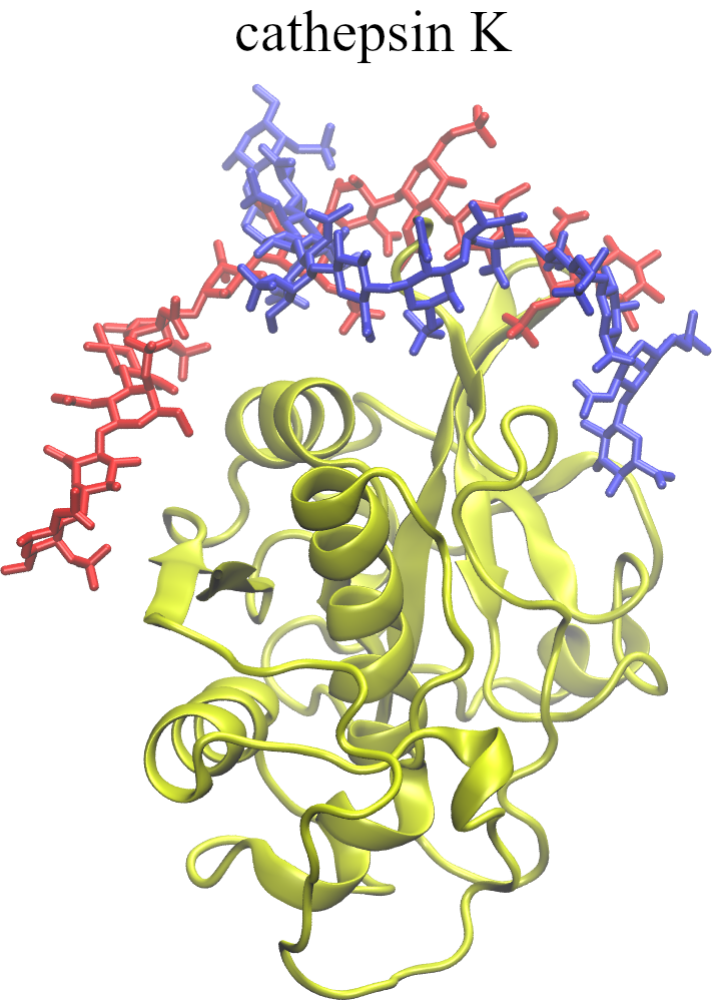
Graphical representation of the ligand 4 starting (in red, licorice) and final (in blue, licorice) position in regard of binding site of cathepsin K (in yellow, new cartoon).

**Figure 6:**
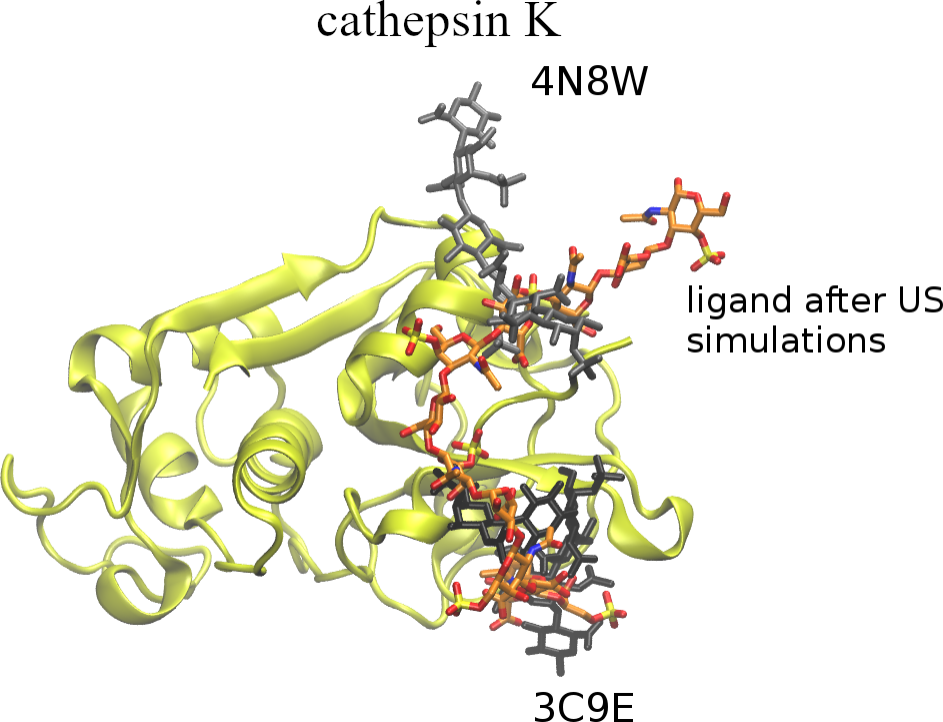
Comparison of the last frame of the US simulation (orange, licorice) with the chondroitin sulfate ligands from the original crystal structures: 3C9E (ligand in black licorice, at the bottom of the figure) and 4N8W (ligand in grey licorice, at the top of the figure).

During the pulling away of the GAG RMSD slowly and steadily increased. During pulling in RMSD only lowered slightly reaching 20 Å which suggests that at the end of US the GAG did not return to a pose similar to the starting one. Relaxation MD neither improved the final conformation. The energy of the system increased from −120 kcal/mol to values between −50 and −40 kcal/mol around the 22nd window and then remained at this level to the end of pulling away. On the way back of the GAG to the binding site the energy slowly decreased and reached −80 kcal/mol at the end of pulling in. During the relaxation MD the energy decreased further to −110 kcal/mol which is almost the same value as observed at the starting point suggesting that this significantly different pose is almost as stable as the initial one. The number of native contacts lowers from ≈1500 to 0 after the 25th window of the US simulation. During pulling in some native contacts are being restored but the number greatly varies and never surpassed 500 contacts. The number of H-bonds during pulling in are also lower than in the initial pose. Visually, the final pose is significantly different than the starting one (Figure 5 and Figure 6). In the binding site the part with the 4-sulfation of the final GAG conformation is perpendicular to the starting one, and the part of GAG with the 6-sulfation is close to the second GAG binding site of the cathepsin K. The final pose of the GAG partially overlapped with both experimentally known binding sites. This is most likely the reason why the energy at the start and at the end of US is similar to the one of the initial pose despite the fact that much a smaller part of the GAG is located at the first binding site. Hence a comparison of the binding position of the ligand with both crystal structures (3C9E and 4N8W) were carried out to reveal which binding site is preferred upon the reassociation of the ligand (Figure 6). It can be seen that the binding of the ligand to the protein at the end of the sliding in represents a combination of both the binding positions from the crystal structures and the RMSatd score obtained for the two different crystal structures are 4.1 Å and 8.3 Å for 3C9E and 4N8W complexes, respectively. The dodecamer ligand docked to the protein in such a way that the hexameric part with the 4-sulfation (as observed in the crystal structure) occupied the 4N8W site and the hexameric part with the 6-sulfation bound to the site observed in the 3C9E structure. The comparison of the final structure and that obtained after the docking yielded RMSatd of 10.2 Å, which shows that the pulling back results in the structure more similar to that of the crystal structure than to the initial docked pose. A similar comparison of the final structure obtained at the end of the sliding in process was done for the other complexes with their corresponding crystal structures and the one obtained after docking. The ligands’ RMSatd values (Tables S1 and S2 in Supporting Information File 1) show that in the majority of the complexes the final structure is more similar to the crystal structure than to the initial docked structure. However, the goal of the study was to compare the final structures to the starting positions rather than to the experimental ones (although both docked and experimental poses are close to each other, see Figure S1 in Supporting Information File 1) to check specificity of the GAG interactions and evaluate the quality of the information obtained from the US simulations.

### Investigation of the protein–GAG recognition in the proximity of the native binding pose

**Figure 7:**
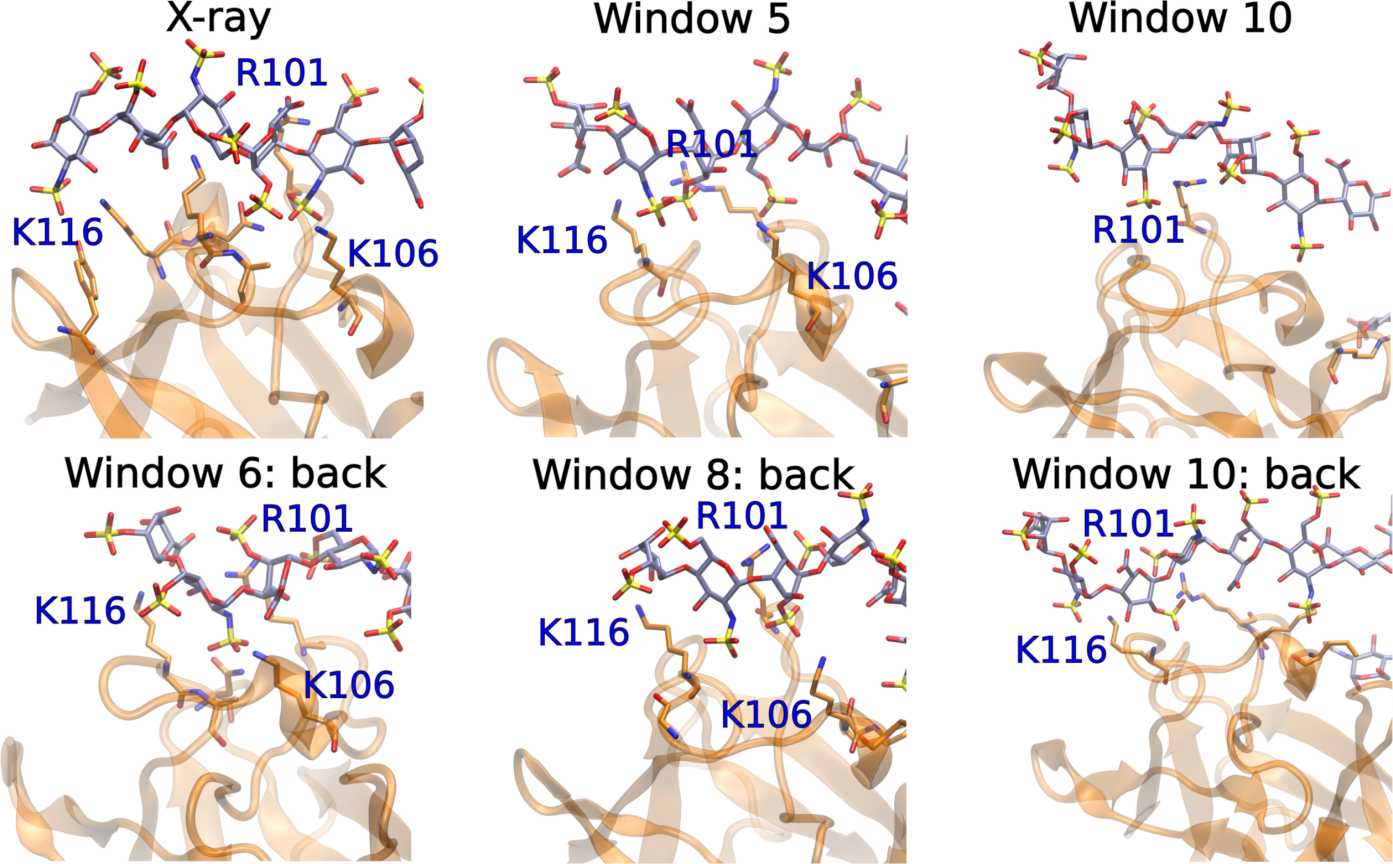
The X-ray conformation of bFGF-HP dp6 (PDB ID: 1BFC) and windows 5 and 10 and windows 6, 8, 10 in the forward and reverse processes for bFGF and ligand 1, respectively. The protein is in cartoon, the GAG and the protein residues establishing H-bonds with the GAG with the occupancy higher than 20% are in sticks. R101, K106, and K116 are labeled.

Next, we analyzed if the US approach is able to reproduce the native binding pose when pulling away a ligand by just a disaccharide unit and returning it back to the binding site. These simulations involved approximately a 10 Å shift from the native complex of ligand 1 from the basic FGF and allowed to investigate the near-native free energy landscape and the respective atomistic details of the protein–GAG recognition. In the forced dissociation process, the RMSD curve looks similar to the ones from the previously described, longer pulling away trajectories: the RMSD values increase gradually as the ligand pose gets closer to the native pose within the shift of a monomeric unit (first part of the pulling away step, 0.5 μs), yielding a rugged shape of the curve (Figure 7). Interestingly, on the way back, the RMSD values reach minimal values at windows 5, 7, and 8, corresponding to the reaction coordinate values of 5 Å, 3 Å, and 2 Å, respectively (as well as corresponding to the 0.5, 0.7 and 0.8 μs of US, respectively), but then go up at the end of the pulling in process.

This is also reflected by the MM/GBSA binding energy analysis (Supporting Information File 1, Figure S7), where the energy gradually increased in the process of dissociation with the exception of the stabilized conformation at window 5 (0.5 μs), where the binding strength is energetically comparable with the one of the native binding pose. The MM/GBSA free energy landscape is very rugged on the way of pulling the ligand in. However, the most favorable energies of very comparable values are observed in windows 6 and 8 (0.6 and 0.8 μs, respectively) suggesting that while for window 8 the proximity to the native pose was energetically favorable, the interaction free energy minimum in window 6 corresponds to a distinct non-native binding pose. This suggests a high heterogeneity of the free energy landscape in the proximity of the native binding pose and a high propensity for multipose binding in the system. The number of native contacts and total H-bonds gradually decrease in the dissociation process (Figure 7), while there is a clear peak of the non-native contacts number and additional H-bonds at window 5 suggesting a partial stabilization of the binding by H-bonds, also in agreement with the MM/GBSA binding energy trend. On the way back, at window 8, the ligand establishes most of the native contacts which also correspond to the increase of the number of H-bonds established. This points out that several stabilized and energetically comparable binding poses co-exist in the system. This is also supported by the absence of significant correlations between the MM/GBSA energy and RMSD to the initial pose (Supporting Information File 1, Figure S8).

Another way to calculate the free energy landscape with less details but in a manner more sound for the applied protocol, is to estimate PMF along the reaction coordinate. Such calculations also support the conclusion that the applied protocol did not succeed in returning the system to the original binding pose (Supporting Information File 1, Figures S9–S11). In comparison to the data from the MM-GBSA approach, the PMF data show even larger differences between the starting and the final poses obtained in the US trajectories.

In turn, results of the analysis of pairwise correlations between the number of established H-bonds, MM/GBSA free energy, native, non-native and total number of contacts differ for pulling out and pulling in processes (Table 3). For the dissociation, as expected the number of native contacts and H-bonds correlate very well with the MM/GBSA energies (Pearson correlation coefficients obtained for all frames of the trajectories are 0.81 and 0.76, respectively), while on the way back, the still high correlation with the H-bond number (0.58) and an essentially decreased one with the native contact number (0.36) mean that hydrogen binding dominates the binding energetics of the system and is an origin of multipose binding.

**Table 3:** Pearson correlation coefficients obtained for all frames of the pulling away and pulling in MD trajectories between the protein–GAG recognition parameters. Native: native contacts; non-native: non-native contacts; total: the sum of native and non-native contacts.

	Pulling away					Pulling in				
	N_native_	N_non-native_	N_total_	N_H-bonds_	ΔG_MM/GBSA_	N_native_	N_non-native_	N_total_	N_H-bonds_	ΔG_MM/GBSA_

N_native_	–	–	–	0.65	0.81	–	–	–	0.18	0.36
N_non-native_	–	–	–	0.02	0.08	–	–	–	0.25	0.34
N_total_	–	–	–	0.54	0.72	–	–	–	0.36	0.58
N_H-bonds_	0.65	0.02	0.54	–	0.76	0.18	0.25	0.36	–	0.57
ΔG_MM/GBSA_	0.81	−0.08	0.72	0.76	–	0.36	0.34	0.58	0.57	–

When correlating the values for MM/GBSA and the number of H-bonds averaged per each US window, the correlation coefficients for the pulling away and pulling back processes are 0.97 and 0.56, respectively. Despite these significant differences in the correlations, implying a more complex free energy landscape topology when the ligand is pulled in, the energies per H-bond calculated from the linear regression model are very similar: −10.1 ± 0.6 kcal/mol and −10.5 ± 1.0 kcal/mol for pulling away and in, respectively. These differences in the correlations, however, can be partially attributed to the arbitrary choice of the US reaction coordinate which can affect the pulling away and pulling in processes and, therefore, the data described here.

Furthermore, we analyzed in detail the most representative H-bonds (with the occupancy higher than 20%) established at different US windows that were the most distinguishable in the pulling away and pulling in processes. In particular, we analyzed the X-ray conformation (PDB ID: 1BFC) based MD trajectory and windows 5 and 10 and windows 6, 8, 10 in the forward and reverse processes, respectively (Figure 7). Interestingly, in all these windows with more favorable binding energies, particular three positively charged residues, R101, K106, and K116, maintained strong H-bonds that have been also established in the X-ray structure-based MD simulation [51]. Some of these residues are absent as the most contributing to H-bonding in the less stable complexes (both last windows of the pulling away and pulling in processes). At the same time, the essential difference between the H-bonding pattern observed in the unrestrained MD simulation of the X-ray structure is that there were several non-charged residues (N8, A17, Y84) among the top residues contributing to H-bonds, while almost exclusively positively charged residues were observed to be substantial H-bond contributors in the US windows. In a microsecond-scale MD simulation of the same X-ray structure, three non-charged residues were identified as the top MM/GBSA free energy contributors [52]. This suggests that despite a very complex free energy landscape in the proximity of the native pose, the native pose can be potentially distinguished by the essential contributions of the non-charged residues to the GAG recognition. Further, this implies a certain degree of specificity and not simply electrostatics-driven interactions in this particular molecular complex. Estimation of the free energy barriers in the completed analysis suggests that the sliding of a long GAG on the protein surface is a feasible process that could underline the natural recognition of the specific GAG patterns by a protein target.

## Conclusion

In this study, the US approach was used to pull away a GAG ligand from the binding site and then to pull it back in to the binding site. The goal was to analyze if US is able to reproduce experimentally obtained structures, and if it can contribute to a deeper understanding of GAG properties as protein recognition specificity and multipose binding. Although the US is a powerful method it was shown not to be able to accurately reproduce experimental structures or the most energetically favorable binding poses in the majority of the investigated systems with the particular protocols we applied in this study. The limitations in our study can be attributed to two main factors. Firstly, the relatively short sampling times (100 ns) may have been insufficient to adequately equilibrate the systems, especially given their complex free energy landscape in each US window. Secondly, the selected reaction coordinates for pulling away and pulling in may not inherently suggest unique reversible pathways. To improve the convergence and sampling of the free energy landscape, advanced sampling protocols can be employed or the US simulations can be repeated multiple times. Therefore, the data obtained in this study and the conclusions related to these data are rather qualitative. In the next steps, we plan to apply more advanced sampling protocols. However, even when using the described protocol in some of the systems it was able to bring the ligand back to the binding site (in two cases with comparable accuracy to one of the most powerful GAG docking tools (RS-REMD), which corresponds to RMSD values <4 Å). Additionally, it allowed to observe multipose binding phenomena manifesting other energetically favorable binding poses of the GAG in the binding site. In these cases, although the RMSD values with reference to the experimental structures were high as only a very small part of the final GAG binding pose overlapped with the initial structure, binding energies remained almost at the same level as the ones corresponding to the experimental binding poses. Regarding the specificity, in most cases a partial overlap between the GAG parts in the experimental and the pulled in structures corresponding to the same sulfation pattern/amount was observed. Nevertheless, in one of the simulations of the basic fibroblast growth factor system a less sulfated part contributed comparably to the sulfated one suggesting a potential of non-purely electrostatics dominance in the protein–GAG interactions. The more detailed analysis of the GAG recognition in this system in near-native states points out to the complexity of free energy landscape but at the same identifies the key charged H-bonding contributors to the GAG binding that together with several non-charged residues in the binding interface potentially determine the specificity of the interactions in this complex. The analysis of free energy landscapes in the studied systems suggests that sliding of a GAG along a binding site in a protein target could occur naturally and, therefore, could be a way for a protein to effectively sample different particular GAG recognition patterns. The findings in this work should contribute to the broadening of the knowledge regarding the specificity of protein–GAG interactions and the limitations of the computational tools employed to analyze them.

## Supporting Information

File 1 Additional information and graphical representations.
