# Supplementary material for: Studying specificity in protein–glycosaminoglycan recognition with umbrella sampling
Source: Beilstein J Org Chem. 2023 Dec 19;19:1933–46. doi: 10.3762/bjoc.19.144 (PMC10760462; doi:10.3762/bjoc.19.144)
Supplement: File 1 — Additional information and graphical representations. [file Beilstein_J_Org_Chem-19-1933-s001.pdf]

BEILSTEIN JOURNAL OF ORGANIC CHEMISTRY

Supporting Information

for

Studying specificity in protein–glycosaminoglycan recognition with umbrella sampling

Mateusz Marcisz, Sebastian Anila, Margrethe Gaardløs, Martin Zacharias and Sergey A. Samsonov

Beilstein J. Org. Chem. **2023,** *19,* 1933–1946. doi:10.3762/bjoc.19.144

Additional information and graphical representations

License and Terms: This is a supporting information file under the terms of the Creative Commons Attribution License (https://creativecommons.org/licenses/by/4.0). Please note that the reuse, redistribution and reproduction in particular requires that the author(s) and source are credited and that individual graphics may be subject to special legal provisions.
The license is subject to the *Beilstein Journal of Organic Chemistry* terms and conditions: (https://www.beilstein-journals.org/bjoc/terms)

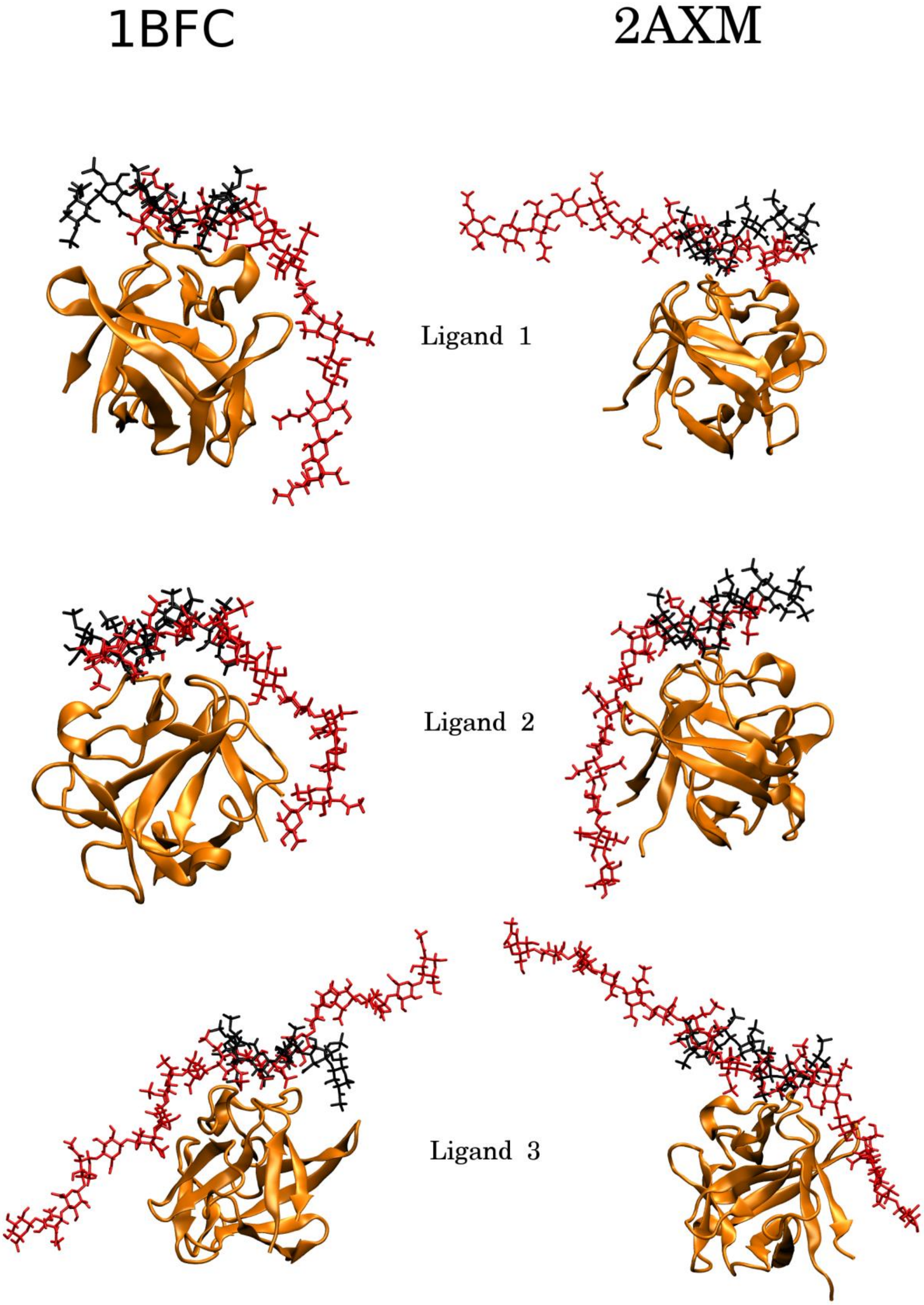

Figure S1. Graphical representation of ligands docked using RS-REMD method (licorice, in red) in comparison to the experimental structure (licorice, in black).

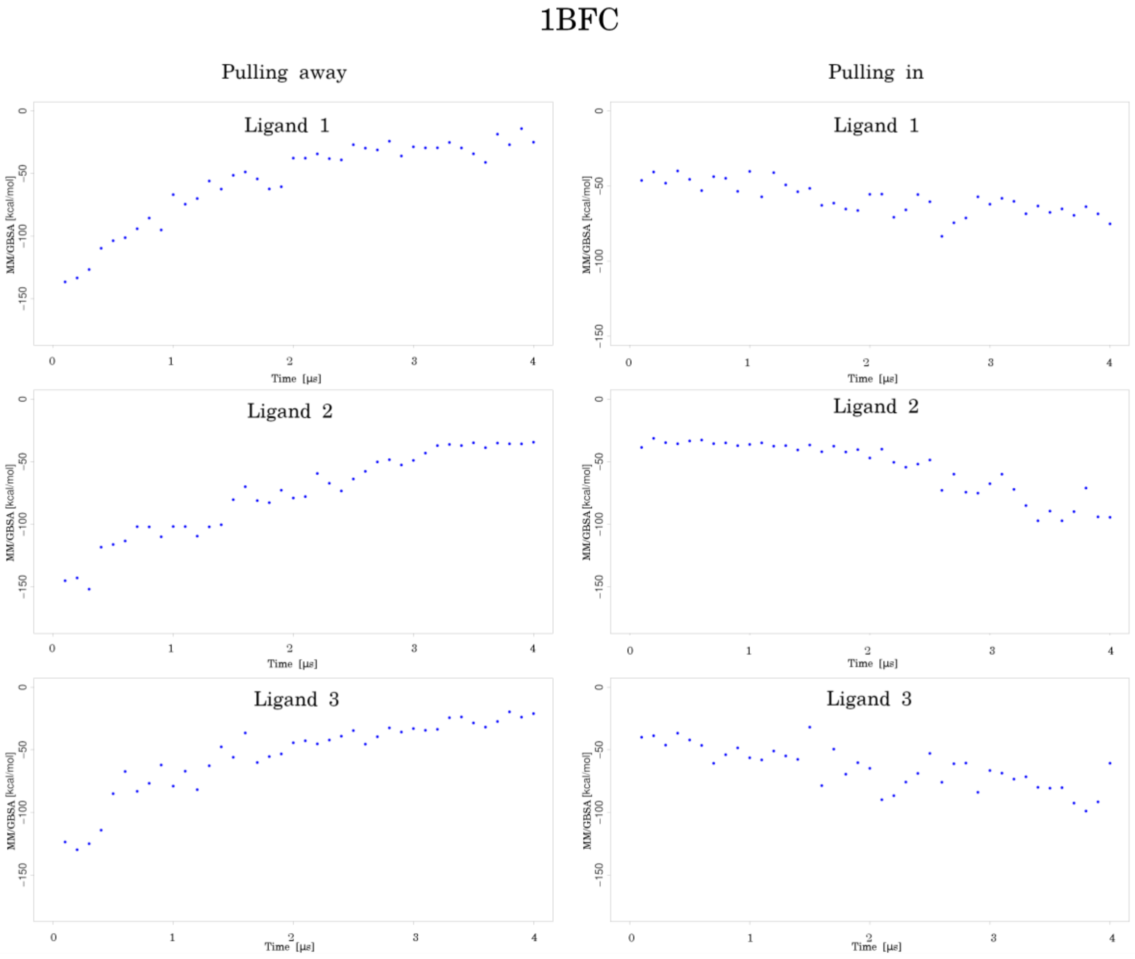

Figure S2. Energies obtained from MM/GBSA analysis for 1BFC complexes with 3 different ligands: ligand is pulled away from the binding site (left panel) and is pulled in towards the binding site (right panel).

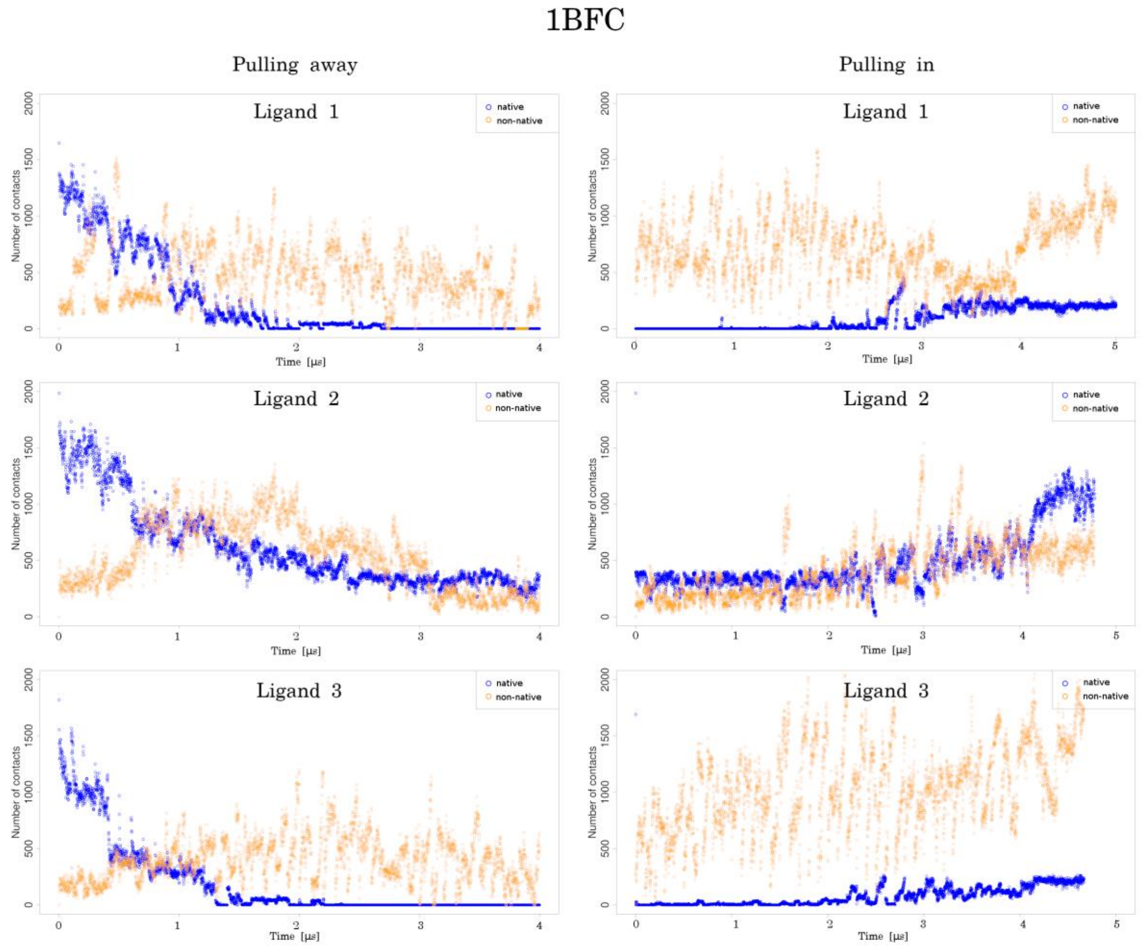

Figure S3. Number of native and non-nonative contacts obtained using cpptraj script from AMBER suite for 1BFC complexes with 3 different ligands: ligand is pulled away from the binding site (left panel) and is pulled in towards the binding site (right panel).

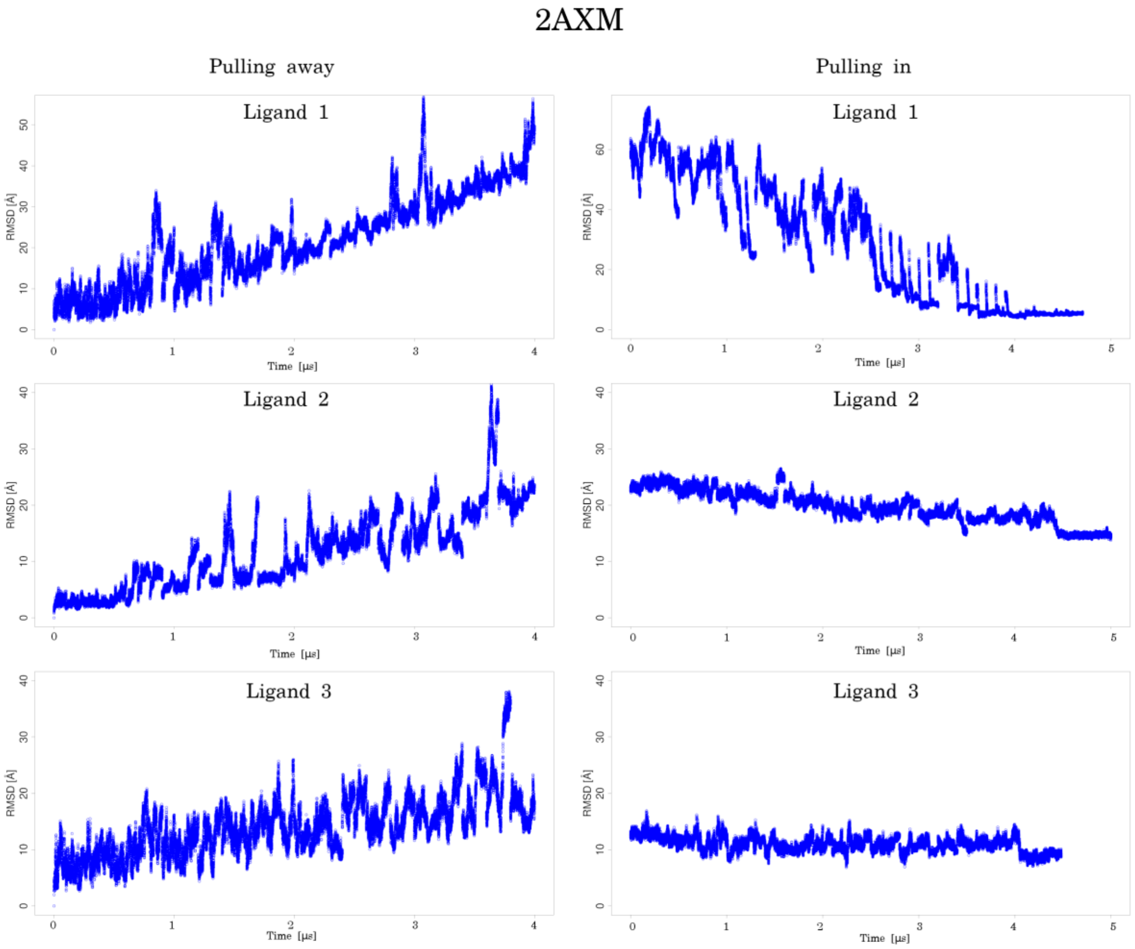

Figure S4. RMSD values obtained using cpptraj script from AMBER suite for 2AXM complexes with 3 different ligands: ligand is pulled away from the binding site (left panel) and is pulled in towards the binding site (right panel).

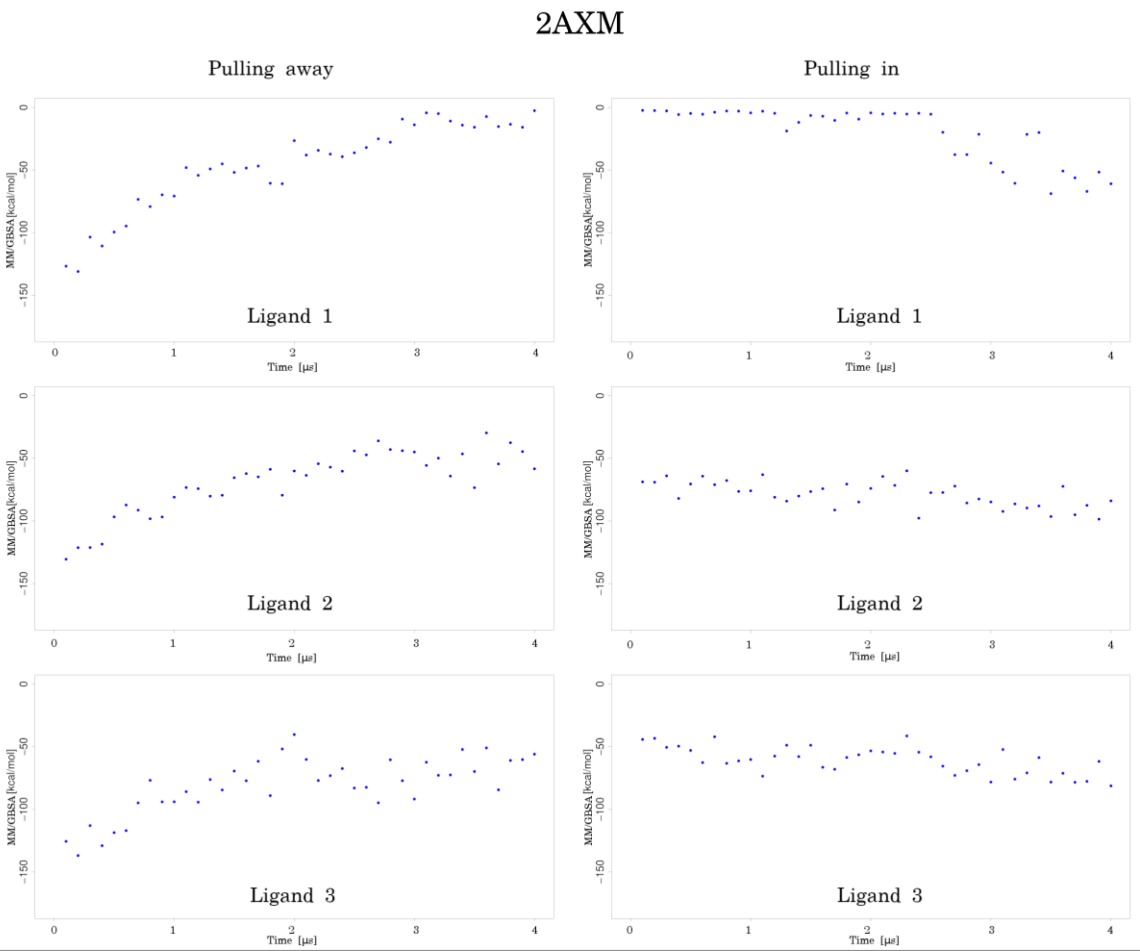

Figure S5. Energies obtained from MM/GBSA analysis for 2AXM complexes with 3 different ligands: ligand is pulled away from the binding site (left panel) and is pulled in towards the binding site (right panel).

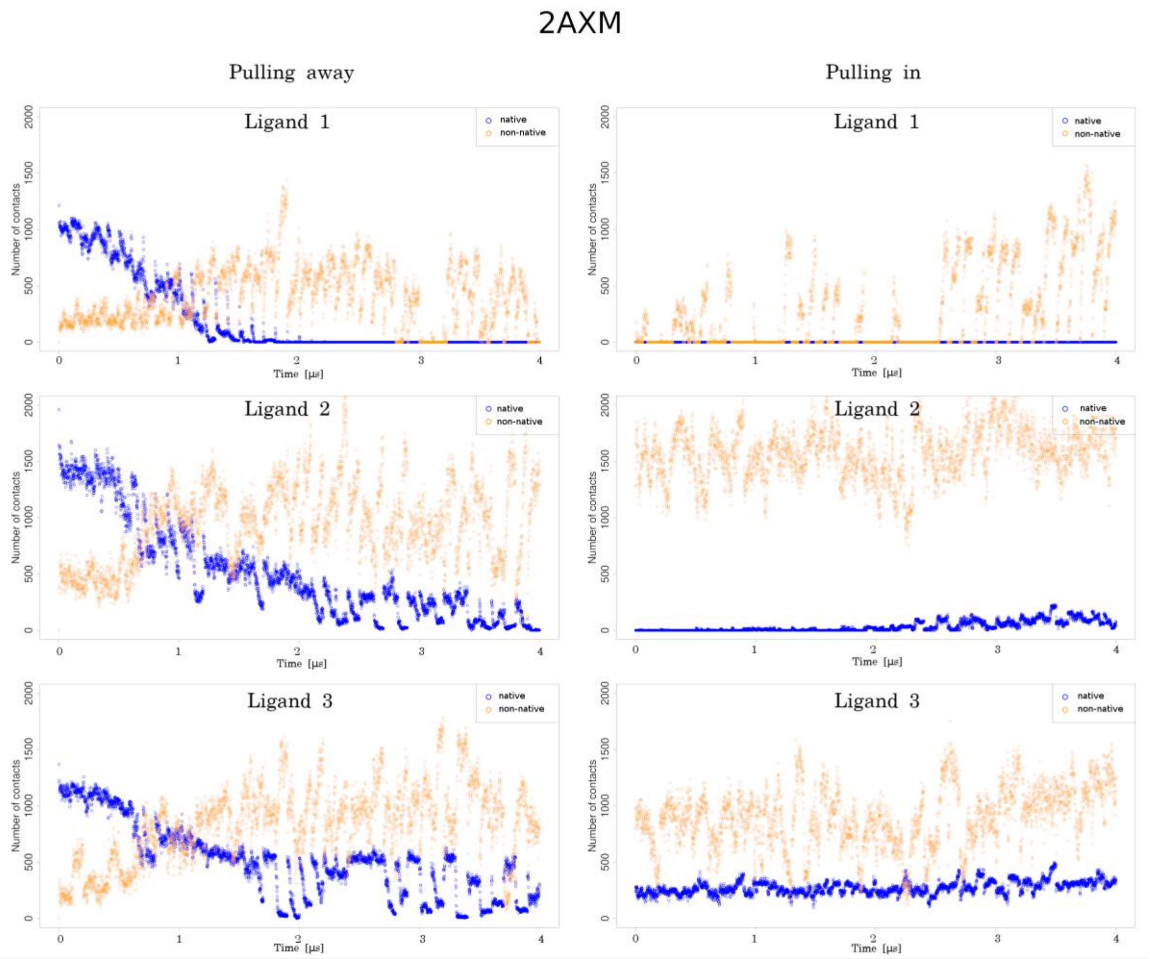

Figure S6. Number of native and non-native contacts obtained using cpptraj script from AMBER suite for 2AXM complexes with 3 different ligands: ligand is pulled away from the binding site (left panel) and is pulled in towards the binding site (right panel).

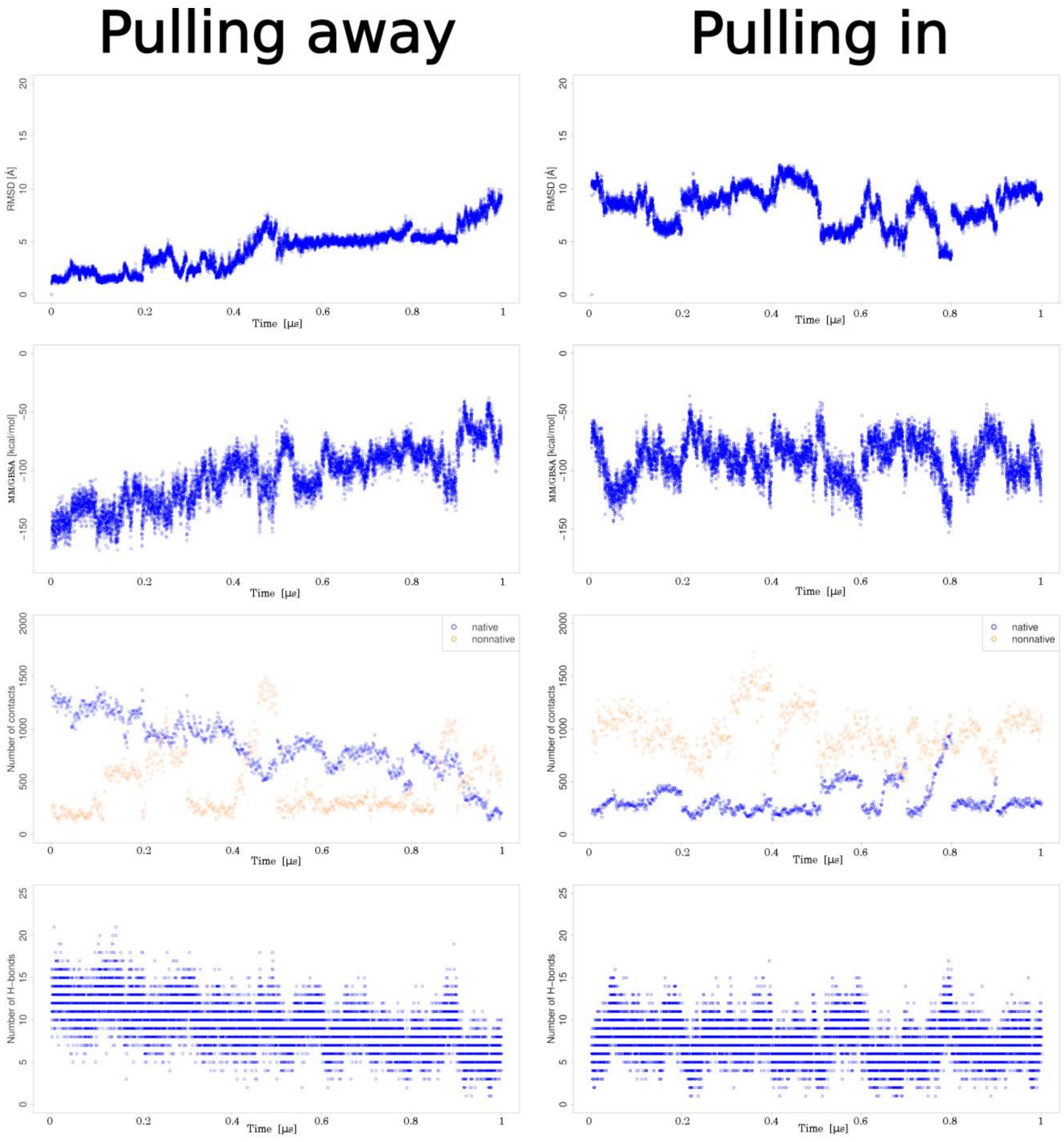

Figure S7. Pulling away and pulling in ligand 1 by dp2: RMSD, MM/GBSA binding energy, established contacts and h-bonds number.

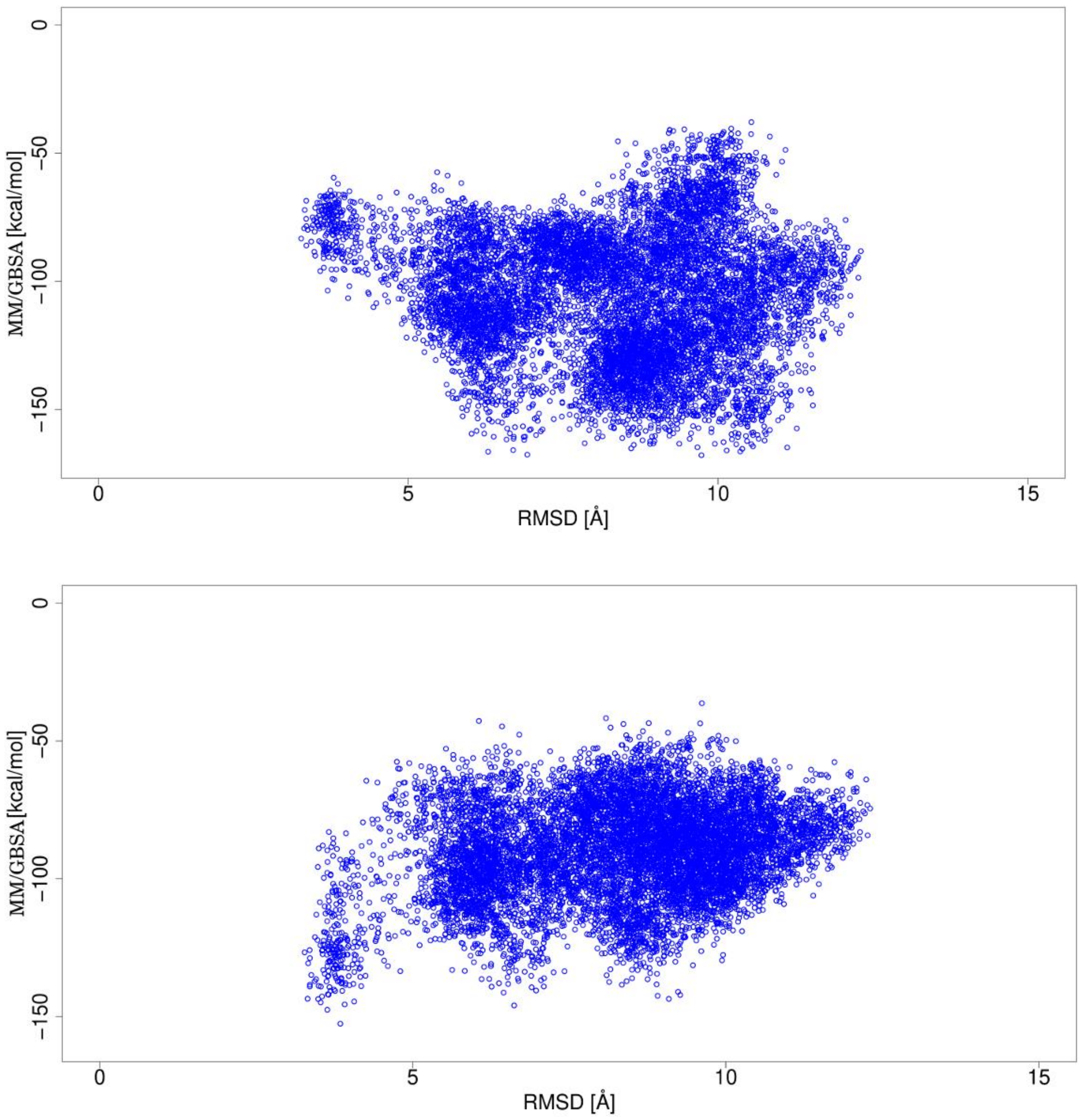

Figure S8. MM/GBSA energy vs. RMSD for the pulling away (upper panel) and pulling in (bottom panel) ligand 1 by dp2. The Pearson and Spearman correlation coefficients are −0.18, −0.16 and 0.25, 0.20, respectively.

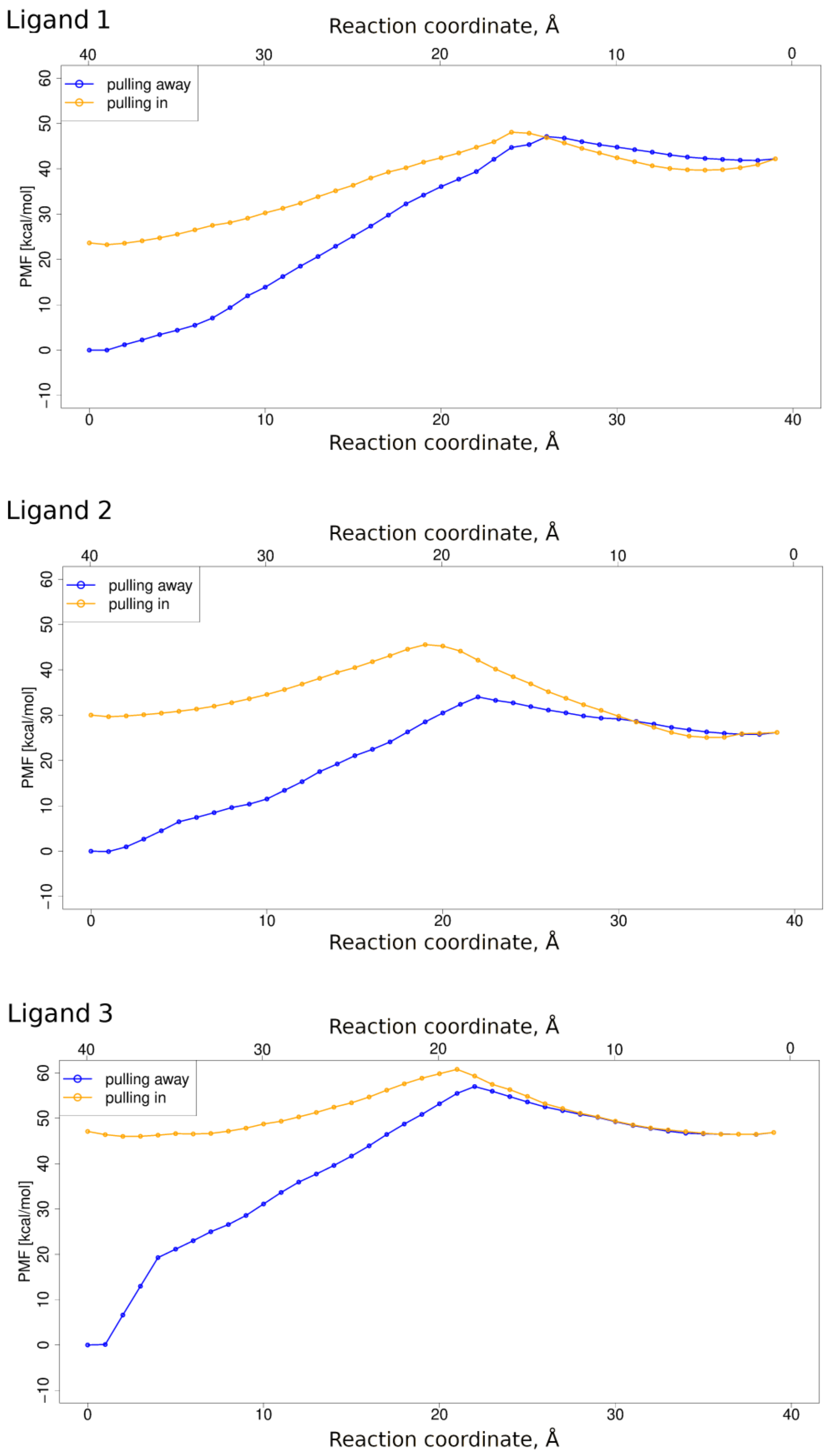

Figure S9. PMF of pulling away (the axis is at bottom) and pulling in (the axis is at top) along the reaction coordinate for ligands 1, 2, and 3 for 1BFC.

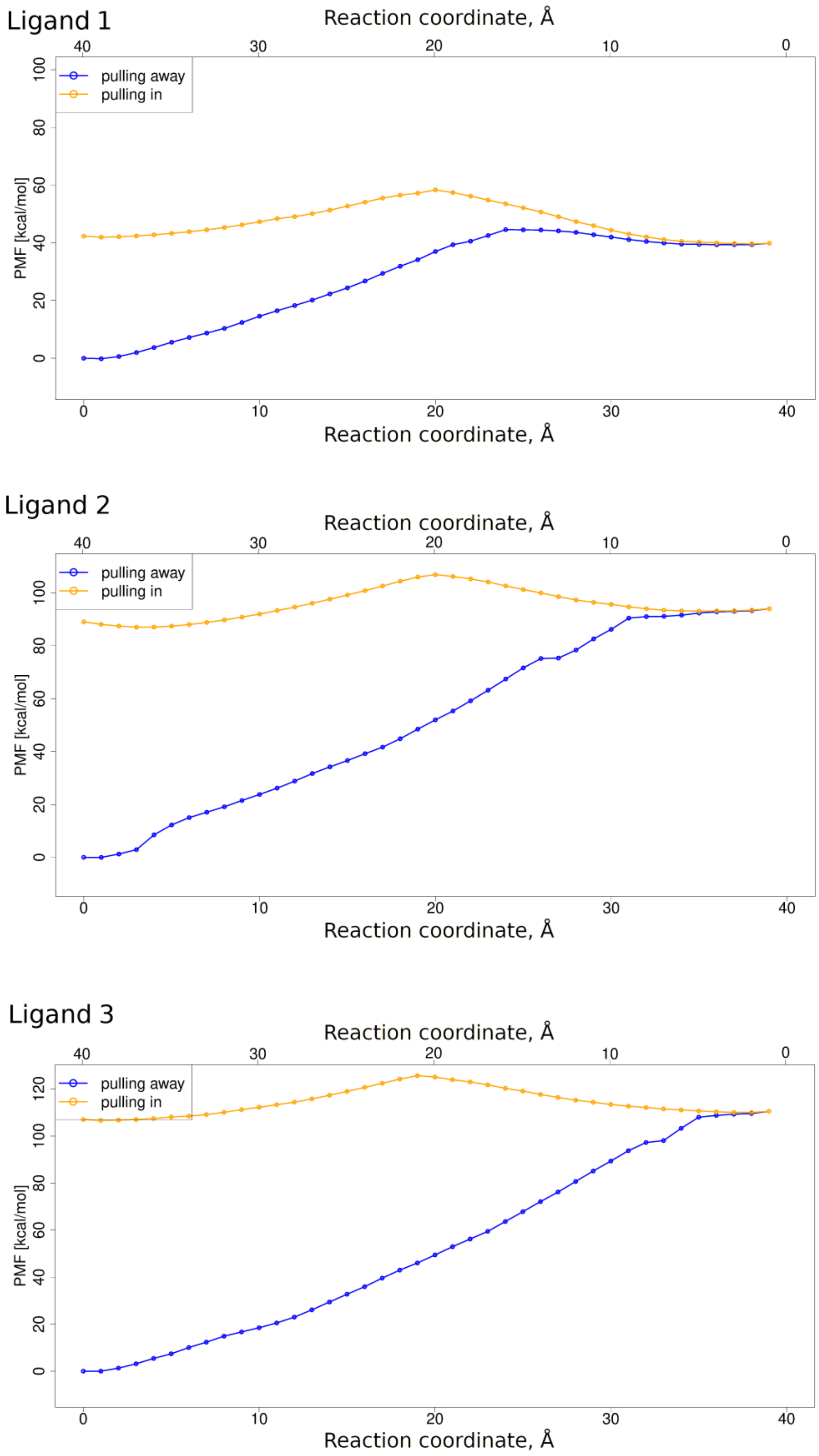

Figure S10. PMF of pulling away (the axis is at bottom) and pulling in (the axis is at top) along the reaction coordinate for ligands 1, 2, and 3 for 2AXM.

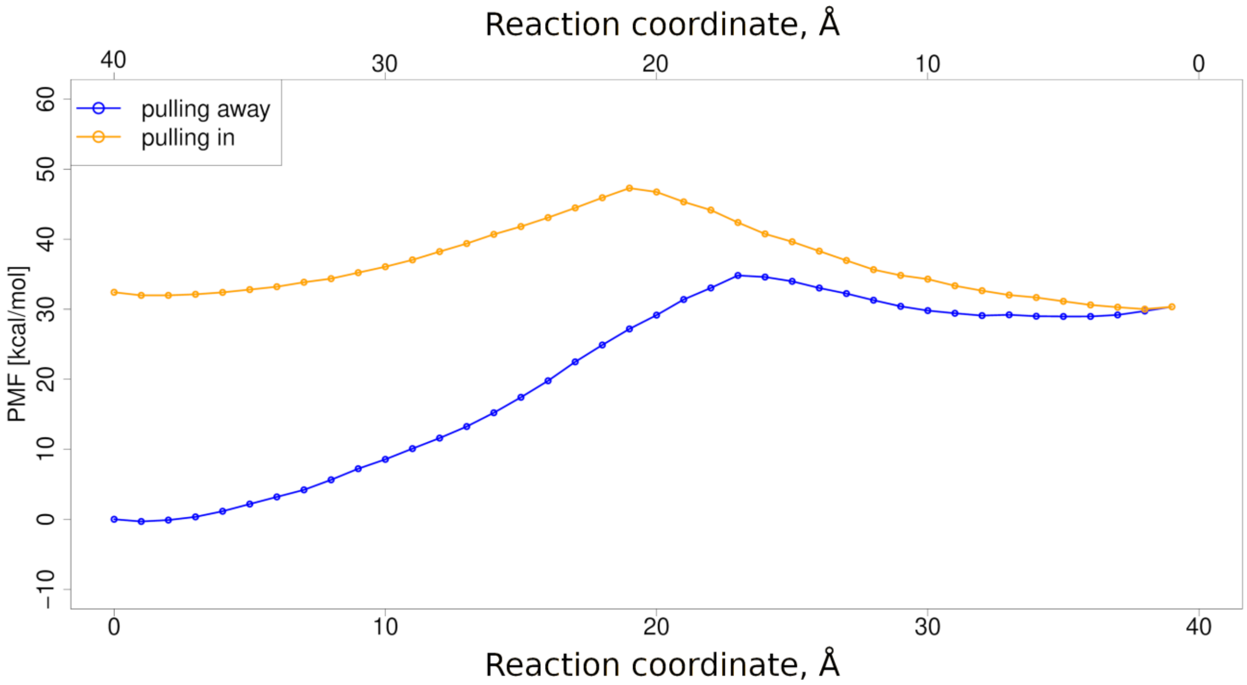

Figure S11. PMF of pulling away (the axis is at bottom) and pulling in (the axis is at top) along the reaction coordinate for ligand 4 for 3CE9.

Table S1. Ligand's RMSatd in Å showing comparison of the last frame in the pulling in process with their corresponding experimental structure.

Protein	Ligand	RMSatd score
1BFC	Ligand 1	9.3
	Ligand 2	6.6
	Ligand 3	7.2
2AXM	Ligand 1	4.1
	Ligand 2	4.3
	Ligand 3	7.7
3C9E	Ligand 4	4.1
4N8W	Ligand 4	8.3

Table S2. Ligand's RMSatd in Å showing comparison of the last frame in the pulling in process with their corresponding docked structure.

Protein	Ligand	RMSatd score
1BFC	Ligand 1	15.8
	Ligand 2	3.2
	Ligand 3	14.5
2AXM	Ligand 1	3.8
	Ligand 2	9.4
	Ligand 3	18.0
3C9E	Ligand 4	10.2
